# The effect of exercise in patients with lower limb lymphedema: a systematic review

**DOI:** 10.2340/1651-226X.2025.42560

**Published:** 2025-03-31

**Authors:** Merete Celano Wittenkamp, Jan Christensen, Anders Vinther, Carsten Bogh Juhl

**Affiliations:** a Department of Physiotherapy and Occupational Therapy, Copenhagen University Hospital, Herlev and Gentofte Hospital, Denmark; b Department of Sport Science and Clinical Biomechanics, University of Southern Denmark, Odense, Denmark; c Department of Occupational Therapy and Physiotherapy, Copenhagen University Hospital – Rigshospitalet, Denmark; d Department of Clinical Medicine, University of Copenhagen, Denmark

**Keywords:** lower limb lymphedema, physical activity, health-related quality of life, adverse events

## Abstract

**Purpose:**

To summarize the evidence of the immediate and long-term effect of exercise interventions in patients with either primary or secondary lower limb lymphedema (LLL) on health-related quality of life (HR-QOL), physical function, self-reported symptoms, lower limb volume, and adverse events.

**Design:**

Systematic review following the guidelines from the Cochrane Handbook of Systematic Reviews of Interventions.

**Data sources:**

MEDLINE, EMBASE, CINAHL, Cochrane Central Register of Controlled Trials (CENTRAL), and clinicaltrials.gov.

**Eligibility criteria:**

Prospective exercise trials investigating exercise interventions as a single- or multicomponent programme in patients with LLL including assessment of at least one of the following outcomes: HR-QOL, self-reported LLL symptoms (heaviness, tension, and pain), physical function, or lower limb volume. Randomized controlled trials (RCTs), single-group studies, and cross-over trials were eligible. Trials with participants at risk of LLL or a diagnosis of filariasis or lipedema were excluded.

**Results:**

Twelve studies were included: three RCTs, five single-group studies, and four cross-over trials with a total of three hundred and sixty-seven participants. In patients with LLL, irrespective of severity, exercise seemed to have small but positive effects on HR-QOL, physical function, pain, and lower limb volume. Quality assessment showed high risk of bias. Large heterogeneity in participants, interventions, and outcome measures hinders performing of meta-analyses.

**Interpretation:**

Based on a small number of studies with large clinical heterogeneity, poor methodological quality, hence low level of certainty of evidence, it was not possible to provide evidence-based recommendations on exercise for patients with LLL.

## Introduction

Lymphedema (LE) is a chronic condition due to impairment in the lymphatic system causing subcutaneous protein-rich edema and inflammation in the affected area [1]. LE can develop due to congenital lymphatic malformations (primary) or acquired damage to the lymphatic system, for example cancer treatment or trauma (secondary LE). In severe cases, deposition of fat and fibrotic tissues, irreversible skin changes, wounds, and cellulitis occur with risk of hospitalization [2]. LE cannot be cured but can be managed by Complete Decongestive Therapy (CDT) consisting of skin care, manual lymphatic drainage (MLD), exercise, and compression therapy [3]. Movement and exercise activate the muscle venous pump and can increase circulation of the lymphatic fluid and thereby reduce symptoms of swelling and improve well-being [1, 4]. Exercise therapy for patients with cancer-related LE is recommended by the latest guidelines from the American College of Sports Medicine (ACSM) [5]. Despite recommendations, patients with LE do not necessarily engage in regular exercise due to fear of exacerbation of the LE and lack of knowledge about whether exercise therapy is beneficial [6]. Previously, systematic reviews have summarized the effectiveness of exercise therapy on prevention and treatment of LE reporting exercise to be safe for patients with LE and with a potential to alleviate LE symptoms [7–9]. Two reviews have mainly included exercise trials with patients diagnosed with breast cancer-related lymphedema (BCRL) [7, 8] due to a higher incidence of BRCL in the upper limb compared to other types of LE [10, 11]. However, results from exercise trials on patients with BCRL may not apply to patients with lower limb lymphedema (LLL). The review by Hsu et al. included trials exclusively on patients with LLL after gynecological cancer and focused on the outcomes: limb volume and self-reported symptoms [9]. A systematic review should include meaningful outcomes to the intended users according to the Cochrane Handbook of Systematic Reviews of Interventions [12]. LE may have a negative impact on physical function and health-related quality of life (HR-QOL) [6, 13]. Therefore, a need for a systematic review with a broader selection of outcomes of interest including adverse events and with the inclusion of patients with either primary and secondary lower limb LE seems relevant as the physiological response to exercise is expected to be similar among primary and secondary LLL. Hence, the aim of the current study is to summarize the evidence of the immediate and long-term effect of exercise therapy measured on HR-QOL, physical function, self-reported symptoms, lower limb volume, and adverse events in patients diagnosed with either primary or secondary LLL.

## Methods

This systematic review was conducted according to recommendations from the Cochrane Handbook of Systematic Reviews of Interventions [12] and was registered in the international prospective register of systematic reviews PROSPERO with the registration number CRD42022340176.

### Databases and search strategy

Four databases were searched with a combination of Medical Subject Headings and free text words: Embase, MEDLINE, CINAHL, and the Cochrane Central Register of Controlled Trials (CENTRAL) databases. No restrictions on publication year or language were applied. The search matrix contained two foci, LLL and exercise therapy, and was used across all databases (full search strategies are presented in Supplementary Appendix 1–4). References of included studies and ClinicalTrials.gov were searched to identify additional unpublished and ongoing studies. Authors were contacted for unpublished completed trials. The final search was conducted July 17, 2024.

### Study selection criteria

Prospective studies investigating the effect of exercise therapy (defined as physical activity that is planned and structured with the goal of improving health-related quality of life (HR-QOL)), LE-related symptoms (heaviness, tension, and pain), physical function, or decreasing the volume of the LE on patients with either primary or secondary LLL were eligible. The exercise intervention could be delivered as a single component intervention or as part of a multi-component intervention in combination with other interventions, for example compression therapy, bandaging, or MLD in participants with unilateral or bilateral LLL. Randomized controlled trials (RCTs) comparing exercise therapy with no exercise, usual care, or patient education were included. Also, single group studies cross-over with reported pre- and post-assessments were included due to an expectation of a low number of RCTs. Cross-over trials were included to assess immediate effects of exercise on LE-related symptoms and volume. Studies with participants at risk of developing LLL or participants with both upper and lower limb LE were not eligible, unless separate data were reported for patients diagnosed with LLL. Furthermore, trials with participants with LLL caused by filariasis or lipedema were excluded.

### Outcomes

The primary outcome of interest was the evaluation of HR-QOL in the longitudinal studies. Secondary outcomes were physical function, for example 6 Minute Walk Test and 30 s Sit to Stand Test, and self-reported LLL symptoms: heaviness, pain measured on a Visual Analog Scale (VAS) or Numeric Rank Scale (NRS), and post-exercise change in lower limb volume, assessed by non-imaging tools (e.g. circumferential measurements, perometry, and water displacement). Outcomes of interest in the cross-over trials were self-reported symptoms and lower limb volume. Adverse events, for example cellulitis, wounds, and muscle injuries, were also included, if reported.

### Study selection

Records identified in the search were imported into the reference programme, Covidence.org; duplicates were removed before screening of titles and abstracts. Two reviewers independently screened titles and abstracts for eligibility. Decision was reached by consensus. Articles were retrieved for full text reading and was evaluated for eligibility by two reviewers independently. Final decision was reached by consensus. One reviewer extracted data to an Excel Spreadsheet. Data extraction was crosschecked for accuracy by a second reviewer, and disagreements were solved by discussion with a third reviewer. In case of missing data, these were requested from the corresponding author.

### Assessment of risk of bias in the included studies

Risk of bias of HR-QOL, physical function, symptoms, and lower limb volume was assessed independently by two reviewers using the Cochrane Risk of Bias assessment tools for the relevant study designs – Cochrane Risk of Bias Tool 2.0 for randomized trials (RoB 2.0) [14], Cochrane Risk of Bias Tool for randomized cross-over trials [15], and single group studies assessed by Risk of Bias In Non‐randomized Studies of Interventions [16]. Disagreements between reviewers were solved by discussion. Studies considered to have a high risk of bias were not excluded, but the quality ratings were clearly described in the results, and the impact of studies with high risk of bias was evaluated.

### Meta-analysis and overall quality assessment

Meta-analysis for relevant outcomes was intended a priori and described in the PROSPERO registration. However, large clinical heterogeneity in study designs, populations, interventions, and outcome measures hinders pooling of data in a meta-analysis. Hence, data were presented narratively; overall quality rating of the studies by Grading of Recommendations Assessment, Development and Evaluation (GRADE) was performed independently by two reviewers, and consensus was reached by discussion [17].

## Results

### Study characteristics

**Figure 1:**
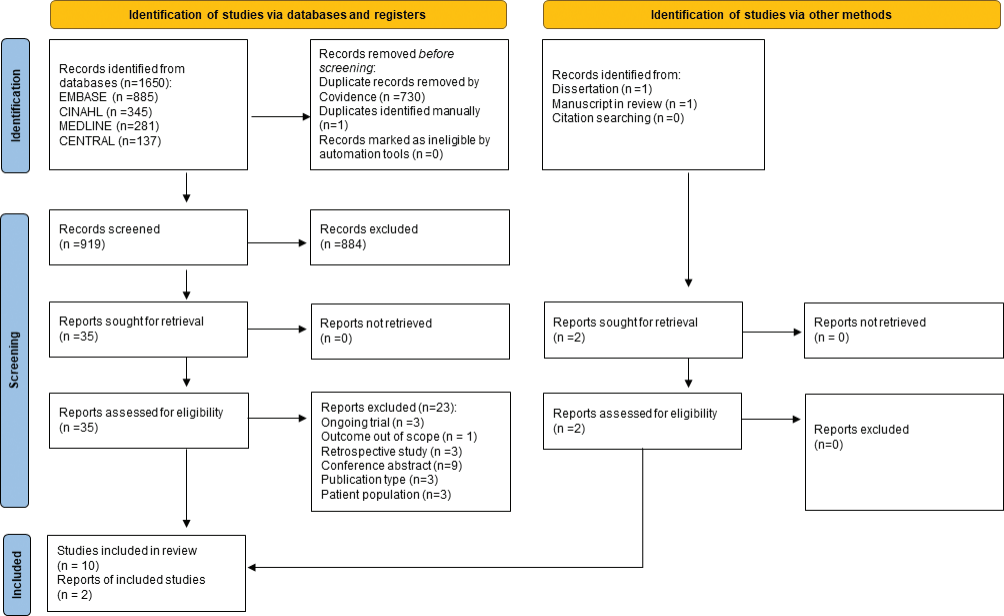
PRISMA 2020 Flow diagram for systematic review that included searches of databases, registers, and other sources.

Ten studies published between 2010 and 2024 were included in the review [18–27] (Figure 1). Additionally, the search in CENTRAL and clinicaltrials.gov identified trial registrations of several unpublished studies, of which results from one dissertation and one manuscript in review were eligible and made available from the authors [28]^1^. The study designs varied with three RCTs [18, 19, 28], five single group studies [20–24], and four crossover trials [25–27]^1^ (Table 1). Sample sizes varied between 9 and 103 participants with a total of 367 participants enrolled. Sixty-seven participants were either lost to follow-up or excluded, leaving a total of 300 participants for the analysis. The participants were mainly diagnosed with cancer-related LLL (*n* = 192) [18–20, 22, 24–29]. Two of these trials also included 33 participants with primary LLL [18, 28]. The remaining two trials did not specify the cause for LLL among participants (*n* =103) [21, 23]. Mean age for analyzed participants across trials ranged from 44.5 to 64.1 years and consisted of 51 men and 249 women. Classification and reporting of severity of LLL varied across studies; three studies included participants with >5%–10% excess limb volume [19, 24, 28], and four studies included participants at stages II–III according to the International Classification of LE from the International Society of Lymphology (ISL) [21, 25, 26]^1^. Among the last studies, participants were classified as early stage LLL [27], mild/moderate/severe LLL [18], and stages II–IV LLL [22]. Two trials did not report on severity of LLL [20, 23] (Table 1). Six additional ongoing studies were identified in clinicaltrials.gov (study characteristics presented in Table 4).

**Table 1:** Study characteristics.

Author Year Country	Aim	Sample size Characteristics Diagnosis Severity, LLL Sex Mean age (SD)	Exercise details	Outcomes
**Randomized Controlled Trials**				
**Do et al.** **2017** **South Korea**	To investigate the effects of a complex rehabilitation program (CRD) in addition to CDT	*n* = 44 Secondary LLL Gynecological cancer >10% excess volume Female only Age: IG: 57.5 (7.7) CG: 55.9 (12.7)	**Intervention group**: CDT + CRD Mode: mixed: stretching, strength exercises with elastic band, 3 sets of 10 repetitions, aerobic exercise on bike and treadmill 15 min each Frequency: 5 sessions per week/4 weeks Delivery: Supervised (week 1–2)/homebased (week 3–4) Intensity: Borg 11–14/64%–76% of predicted max heart rate Duration: 40 min per session Compression: not reported **Control group**: CDT only Frequency: 5 sessions per week/4 weeks Delivery: Therapist-led (week 1–2)/self-MLD and self-bandaging (Week 3–4) Compression: Self-bandaging	**Quality of life** EORTC-QLQ-C30 **Symptoms** Not reported **Physical function** 30s CST-test **Limb volume** Limb volume, calculated from circumferential measures, 4 cm interval,
**Ergin et al.** **2017** **Turkey**	To investigate the effects of aqua lymphatic therapy on unilateral lower extremity lymphedema in the maintenance phase	*n* = 63 Primary LLL (IG 14/CG 13) or Secondary LLL (IG 16/CG 14) Severity Mild: IG 11/CG 12 Moderate: IG 9/CG 10 Severe: IG 10/CG 5 IG: male 6/female 24 CG: male 4/female 23 Age: IG: 44.5 (13.7) CG: 47.7 (16.8)	**Intervention group**: Mode: water-based exercise in 1.4 m deep pool, 32–33, 5°C: breathing, movement chest/trunk, self-massage, movement lower limb, remedial exercises (proximal to distal exercises) Frequency: 2 sessions per week/6 weeks Delivery: Supervised, group based Intensity: low resistance Duration: 45–60 min per session Compression: not reported **Control group**: Self-MLD and remedial exercises Frequency: 2 sessions per week/6 weeks Delivery: Supervised, group based Time: 45–60 min per session Compression: Both groups were asked to maintain usual self-care management during trial	**Quality of life** SF-36 **Symptoms** Not reported **Physical function** 6MWT **Limb volume** Limb volume, calculated from circumferential measures, 5 cm interval + lower leg by WDM
**Jönsson et al.** **2023** **Sweden**	To investigate the efficacy of bicycling exercise at moderate intensity compared to usual daily activity and the feasibility of the exercise in LLL	*n* = 33 Primary or secondary LLL Severity Not reported IG: male 5/female 11 CG: male 5/female 6 Age: IG: 60 (12.6) CG: 71 (12.6)	**Intervention group** Mode: bicycling Frequency: 3–5 times per week/8 weeks Delivery: Unsupervised, homebased Intensity: Moderate (40%–59% of heart rate reserve) heart rate monitor (polar FS 1) Duration: 30–60 min per session Compression: Yes **Control group** Mode: habitual daily physical activity routines or exercise	Quality of life LyQLI Symptoms Not reported Physical function Sub-maximal ergometer test (VO2max) Limb volume Limb volume, calculated from circumferential measures, 4 cm interval
**Single group studies**				
**Angst et al.** **2023** **Switzerland**	To quantify therapy-attributable effects of a comprehensive inpatient rehabilitation program for lower limb lymphedema and to compare the levels of health-related quality of life to population-based norms	*n* = 67 Primary or secondary LLL Severity: ISL stage II 69% Stage III 31% Male 21/female 46 Age: 60.5 (14.8)	Mode: mixed, aquatic training followed by CDT (skin care, MLD, multilayer bandaging) Frequency: 6 sessions per week/3 weeks Delivery: Supervised Intensity: Not stated Duration: 30 min per session (aquatic training) Compression: no compression Instructed in additional MTT (strength training) unsupervised, 30 min per session, not mandatory	**Quality of Life** FLQA-lk SF-36 **Symptoms** Not reported **Physical function** Not reported **Limb volume** Not reported
**Dionne et al.** **2018** **Canada**	To investigate whether patients with lower limb lymphedema can benefit from water immersion exercise training	*n* = 11 Primary or Secondary LLL, gynecological cancer + malignant melanoma Severity not reported Female only Age: 61.9 (12.1)	Mode: water-based circuit training, yoga exercises, aqua-jogging, water bike, aqua step, and trampoline Frequency: 2 sessions per week/6 weeks Delivery: Supervised Intensity: Moderate/vigorous intensity Duration: 45 min per session Compression: not reported	**Quality of life** LyMQOL **Symptoms** Not reported **Physical function** 6MWT **Limb volume** Limb volume, calculated from circumferential measures
**Katz et al.** **2010** **USA**	To assess the feasibility of recruiting and retaining cancer survivors with lower limb lymphedema into an exercise intervention study	*n* = 10 Secondary LLL, urogenital, or gynecological cancer + malignant melanoma >6% excess volume Male 3/female 7 Age: 60.1 (7.7)	Mode: resistance exercise; 12 exercises – seated row, chest press, lateral raises, biceps curl, hip flexion, leg abduction, prone straight leg lift, calf raises + free weights Frequency: 2 sessions per week/5 months Delivery: supervised for 2 months/unsupervised 3 months Intensity: slowly progressive training, weight increased weekly, 2 set to 3 sets of 10 reps/set Duration: 90 min per session Compression: custom fitted compression garment	**Quality of life** SF-36 **Symptoms** Not reported **Physical function** 6MWT + 50ft WT **Limb volume** Limb volume, perometer
**Lindquist et al.** **2015** **Sweden**	To investigate whether exercise can improve lymphedema (and if there is a difference between water-based versus land-based exercise compared to standard care)	*n* = 14* *water = 10 Standard = 3 Land = 1 Secondary LLL Gynecological cancer (+ BCRL, *n* = 55) Severity not reported Female only Age: 58.0 (11.0)^^^ ^^^*n* water = 35 (+BCRL)	Mode: water-based exercise in 1.4 deep pool, 28–29°C: warm up, mobility, strength, slow down mobility, hold/relax, deep breathing. Frequency: 1 session per week/10 weeks Delivery: Supervised **Intensity**: Duration: 50 min per session Compression: during exercise, yes Setting: 3 sites	**Quality of Life** Not reported **Symptoms** Not reported **Physical function** Not reported **Limb volume** Limb volume, WDM or calculated from circumferential measures
**Zeng et al.** **2024** **China**	To investigate the effect of CDT based on fluoroscopy-guided MLD (FG-MLD) combined with Intermittent Pneumatic Compression (IPC) on patients with secondary bilateral LLL after gynecological cancer	*n* = 18 Secondary LLL, Gynecological cancer Severity edema stages II-IV Female only Age: 58.8 (7.2)	Mode: mixed, FG-MLD, IPC 30 min + skin care + multilayer bandaging + functional exercises (walking, stairclimbing, sitting exercises) Frequency: 3–5 times a day Delivery: unsupervised exercise Intensity: not reported Duration: 10–15 min per session, not more than 30 min Compression: multilayer bandaging	**Quality of Life** EORTC-QLQ-C30 **Symptoms** Pain and heaviness **Physical function** Not reported **Limb volume** Circumferential measurements, 6 points at the lower limb
**Cross-over Trials**				
**Abe et al.** **2021** **Japan**	To determine the relative benefits of active exercise with compression therapy (AECT) with different postures for patients with lower limb lymphedema	*n* = 18 Secondary LLL, Gynecological cancer Stage ≥ 2 (staging from ISL) Female only Age: 64.1 (10.8)	Mode: seated or supine exercise on a bike ergometer versus supine position with legs elevated/no exercise Frequency: 3 sessions/1 week apart, randomized order Delivery: supervised Intensity: exercise load determined by Karvonen Formula with exercise intensity at 0.3, 60 revolutions/minute Duration: 15 min per session Compression: newly applied compression bandaging, pressure controlled by Kikuhime pressure sensor All participants were instructed to maintain usual lymphedema self-care management regimen, physical activity level and diet	**Quality of Life** Not reported **Symptoms** Pain & heaviness, VAS 100 mm **Physical function** Not reported **Limb volume** Limb volume, perometry
**Fukushima et al.** **2017** **Japan**	To evaluate the immediate effects of active exercise with compression therapy (AECT) on lower limb lymphedema	*n* = 25 Secondary LLL, Gynecological cancer Stage 2A–2B (staging from ISL) Female only Age: 60.9 (8.3)	Mode: high load/low load at bicycle ergometer versus sitting position/no exercise Frequency: 3 sessions/1 week apart, randomized order Delivery: Supervised Intensity: 10% (high load)/5% (low load) of maximum extension muscular strength versus no exercise Duration: 15 min per session Compression: newly applied compression bandaging, subbandage pressure 40 mmHg at calf, controlled by Kikuhime pressure sensor	**Quality of Life** Not reported **Symptoms** Pain & heaviness, VAS 100 mm **Physical function** Not reported **Limb volume** Limb volume, perometry
**Sierakowski, Piller 2014** **Australia**	To evaluate the effect of sporting compression (SC) on lower limb volume and composition post-exercise in patients with early-stage unilateral secondary lymphedema (and healthy volunteers)	*n* = 9 Secondary LLL (+ healthy subjects, *n* = 9) Early-stage LLL Male 4/female 5 Age 55.6 (8.54) *	Mode: submaximal treadmill exercise test, increased speed or percentage increased every 3 min Frequency: 2 sessions/period between sessions not reported Delivery: supervised Intensity: submaximal intensity Duration: until 85% maximal heart rate reached (220 – age x 0.85) Compression: 1 session with compression tights (SKINS), 19 mmHg at ankle gradually decreasing to 9 mmHg at buttocks/1 session without compression	**Quality of Life** Not reported **Symptoms** Not reported **Physical function** Not reported **Limb volume** Limb volume, perometry
**Wittenkamp** **et al.** **2024** **Denmark** ^1^	To evaluate the safety and possibility of performing High Intensity Interval Training (HIIT) on a stationary bike for participants with cancer-related lower limb lymphedema (LLL) with and without compression garments in a cross-over design	*n* = 21 Secondary LL Stage 1–2B (staging from ISL) 3 male/18 female Age: 52.1 (12.3)	Mode: high intensity interval training Frequency: 2 sessions/1 week apart, randomized order Delivery: Supervised Intensity: high (1 min interval × 7) Duration:23 min per session Compression: 1 session with usual compression garment, 1 session without	**Quality of Life** Not reported **Symptoms** Pain, heaviness, NRS scale 0–10 **Physical function** Not reported **Limb volume** Limb volume. Calculated from circumferential measurements, 8 cm interval

* Mean age and SD were re-calculated by authors. 30s CST: 30 Seconds Chair-Stand Test; 1 RM: 1 repetition maximum; 6 MWT: 6 minute Walk Test; 50ft WT: 50 feet Walk Time; A: Analyzed; BCRL: Breast cancer-related Lymphedema; CG: Control group; CDT: Complete Decongestive Therapy (skin care, manual lymphatic drainage, compression, exercise); D: dropout; EORTC QLQ-C30: European Organization for Research and Treatment of Cancer Quality of Life Questionnaire C30; FG-MLD: Fluoroscopy-guided Manual Lymphatic Drainage; GCLQ-K: Gynecological Cancer Lymphedema Questionnaire – Korean version; HOOS: Hip Osteoarthritis Outcome Score; IG: Intervention group; ISL: International Society of Lymphology; LLL: Lower Limb Lymphedema; MLD: manual lymphatic drainage; PRO: Patient Reported Outcome; RCT: randomized controlled trial; ROM: Range of movement; SF-36: Short Form-36; VAS: Visual Analogue Scale; WDM: Water Displacement Method.

Table 2Result from included trials and risk of bias ratings.

**Table:** 

Author Year Country	Analyzed/Dropout	Results		Adverse events	Authors’ conclusions	Overall Risk of Bias
**Randomized Controlled Trials**						
**Do et al.** **2017** **South Korea**	A = 40 (IG 20/CG 20) D = 4	**HR-QOL – IG** Pre: 60.3 (19.4) Post: 76.6 (32.2) Change Score: +16.3 (SE 4.1) Between groups: *p* = 0.300 **Physical function** **30s CST-IG** Pre: 22.6 (4.7) repetitions Post: 25.0 (3.5) repetitions Change Score: +2.4 (NOT REPORTED) Between groups: *p* ≤ 0.001 **Volume – IG** Pre: 8828.7 (1013.3) ml Post: 8253.2 (876.1) ml Change Score: -575.5 (SE 134.7) ml Between groups: *p* = 0.252	**HR-QOL – CG** Pre: 62.8 (26.9) Post: 69.5 (18.1) Change Score: +6.7 (SE 3.4) **30s CST-CG** Pre: 21.9 (2.3) repetitions Post: 21.5 (2.7) repetitions Change Score: -0.4 (not reported) **Volume – CG** Pre: 8847.3 (1052.5) ml Post: 8208.8 (920.2) ml Change Score: -638.5 (SE 140.7) ml	None	HR-QOL, pain and limb volume improved in both groups. The CR program in the IG showed beneficial effects on muscular strength, physical function, and fatigue without exacerbating LLL	HR-QOL: high risk
						Physical function: high risk
						Volume: high risk
**Ergin et al.** **2017** **Turkey**	A = 57 (IG 30/CG 27) D = 6	**HR-QOL** No overall score reported, but difference between groups were significant (p ≤ 0.05) **Physical function** 6MWT* – IG Pre: 440 (110) m Post: 490 (120) m Change Score: +50 (SE 13.7) m *p* = 0.000 Between groups: not reported Volume* – IG Pre: 11434.7 (4170.9) ml Post: 10726.5 (3841.4) ml Change score: -708.23 (SE 476.2) ml *p* = 0.000 Between groups: *p* =0.7	6MWT* – CG Pre: 435 m (110) Post: 485 m (125) Change score +50 (SE 14.0) m *p* = 0.000 Volume* – CG Pre: 11682.5 (4672.9) ml Post: 11305.2 (4477.3) ml Change score: –377.3 (SE 542.5) ml *p* = 0.000	None	Reduction in limb volume and increase in functional exercise capacity and quality of life were seen in both groups but found to be better in the IG. ALT was a safe effective method for unilateral lower extremity lymphedema during the maintenance phase of CDT	HR-QOL: high risk
						Physical function: high risk
						Volume: high risk
**Jönsson et al.** **2023** **Sweden**	A = 27 (IG 16/CG 11) D = 6	HR-QOL – IG* Pre: 0.7 (0.7) Post: 0.5 (0.7) Change Score: -0.2 (not reported) Between groups: *p* = 0.1 **Physical function** **VO**_**2**_ max – IG* Pre: 2.6 (1.0) Post: 3.0 (0.8) Change Score: 0.4 ( not reported) Between groups: *p* = 0.2 Volume – IG* Pre: 9562 (1434.1) ml Post: 9614 (1371.9) ml Change Score: +52 ( not reported) ml Between groups: *p* = 1.00	HR-QOL – CG* Pre: 0.3 (0.4) Post: 0.4 (0.3) Change Score: 0.1 ( not reported) **VO**_**2**_ max CG* Pre: 2.4 (0.7) Post: 2.6 (0.6) Change Score: 0.2 ( not reported) Volume – CG* Pre: 7939.3 (1100) ml Post: 7824.8 (1012.6) ml Change score: 114.5 (not reported) ml	1 event of >5% excess limb volume	Home-based bicycling exercise at moderate intensity is feasible and improves local tissue water, LE-related disability, physical fitness, and health-related quality of life in persons with LLL. Regular check-ups for volume control and guidance are supportive	HR-QOL: high risk
						Physical function: high risk
						Volume: high risk

**Table:** 

**Single Group Studies**							
**Angst et al.** **2024** **Switzerland**	A = 67 D = 36	**HR-QOL** Pre: 67.8 (15.9) Post: 76.3 (14.2) Change score: 8.5 not reported p-value not reported			Not reported	Those affected by LLL stages II and III benefitted substantially from the intervention, attaining equal or higher levels of HR-QOL than expected compared with the general population norms. Multidisciplinary, inpatient rehabilitation should be recommended for LLL management	HR-QOL: high risk
**Dionne et al.** **2018** **Canada**	A = 7 D = 4	**HR-QOL** Pre: 7.09 (1.8) Post: 8.09 (0.8) Change score: +1 (SE 0.5) *p* = 0.05 **Physical function** **6MWT** Pre: 458.4 (117) m Post: 520.0 (126) m Change score: +61.6 (SE 41.2) m *p* = 0.04	**Volume** Pre: 11417.2 (3400.5) ml Post: 10626.8 (3056.4) ml Change score: – 844.4 (SE 1097.6) *p* = 0.03		Not reported	The exercise programme allows moderate to vigorous intensity for patients with lower limb LE, increases functional capacity and QOL, and does not exacerbate LE	HR-QOL: high risk
							Physical function: high risk
							Volume: high risk
**Katz et al.** **2010** **USA**	A = 10 D = 5* *Dropout from exercise programme, but none lost to follow-up	**HR-QOL** Data not presented, but N.S. **Physical function** **6MWT** Pre: 475 (63) m Post: 504 (66) m Change score + 29 (SE 18.3) m *p* ≤ 0.01	**50s WT** Pre: 11.9 (1.9) seconds Post: 10.5 (1.8) seconds Change score: -1.4 ( not reported) *p* ≤ 0.01 **Volume, affected limb** Pre: 11.383 (5838) ml Post: 11.356 (5794) ml Change score: -27 (SE 1645.0) ml *p* = 0.89		Cellulitis *n* = 2 Muscle injuries *n* = 0	Recruitment of patients with lower limb LE into a 5-month weight-training intervention is feasible. Despite some indication that the intervention may be safe (e.g., a lack of clinically significant interlimb volume increases over 5 months), the unexpected finding of 2 cellulitis infections among participants suggests additional study is required before concluding that patients with lower limb LE can safely perform weightlifting	6MWT: Serious risk High risk
							Physical function: high risk
							Volume: high risk
**Lindquist et al.** **2015** **Sweden**	A = 7 (water) D = 3 Water = 10 Standard = 3 Land = 1	**Volume, affected limb** Pre: 10645 (2190) ml Post: 10819 (2395) ml Change score: + 174 (-172–518 95 % CI) ml *p* = 0.265			Not reported	Survivors with leg LE in the study were too few to draw conclusions, and the HOOS questionnaire is not fully applicable to these patients	Volume: high risk
**Zeng et. Al** **2023** **China**	A = 18 D = 0	**HR-QOL** No overall score reported but change score in all subscales improved *p* ≤ 0.05: Emotional function 25.39 (SE 1.0) Role function 16.44 (SE 0.9) Physiological function 26.00 (SE 1.0) Social function 24.39 (SE 0.7) Cognitive function 23.94 (SE 0.8)		**Pain – scale not reported** Pre: 6 Post: 0 Change score: 6 *p* = 0.007 **Volume of both limbs** Pre: 500.3 (31.9) cm Post: 447 (32.6) cm Change score: -53.3 (SE 3.6) *p* = 0.00	Not reported	CDT based on FG-MLD, combined with IPC, effectively relieves secondary bilateral lower limb lymphedema after comprehensive treatment for gynecological malignant tumors. It also improves subjective symptoms and patients’ QoL, thus deserving clinical reference and promotion	HR-QOL: high risk
							Pain: high risk
							Volume: high risk

**Table:** 

**Crossover Trials**										
**Abe et al.** **2021** **Japan**	A = 18 D = 0	**Pain (mean% change)** Seated: 27.9 (SE 12.5)% Supine: 71.0 (SE 5.3)% CT: 45.4 (SE 6.8)% Seated versus supine *p* = 0.02 Others *p* = N.S. Overall *p* = 0.03	**Heaviness (mean % change)** Seated: 27.5 (SE 15.0)% Supine: 62.3 (SE 6.2)% CT: 57.6 (SE 6.5)% Seated versus supine, *p* = 0.03 Others *p* = N.S. Overall *p* = 0.02			**Volume** **(mean % change)** Seated: -27.9 (SE 12.5)% Supine: -71.0 (SE 5.3)% CT: -45.4 (SE 6.8)% Supine versus CT: *p* = 0.01 Others *p* = N.S. Overall *p* = 0.01		None	An immediate reduction in limb volume was observed after AECT. Supine AECT achieves greater reductions in limb volume and feelings of pain and heaviness than seated AECT	Volume: low risk
										Pain/heaviness: low risk
**Fukushima et al.** **2017** **Japan**	A = 23 D = 2	**Pain** High load: 32.8 (SE 1.9) Low load: 24.1 (SE 1.9) CT: 12.0 (SE 1.8) All groups, *p* = N.S. Overall *p* = 0.22	**Heaviness** High load: 47.2 (SE 1.5) Low load: -3.4 (SE 1.6) CT: 33.5 (SE 1.6) All groups *p* = N.S. Overall *p* = 0.15			**Volume** High load: 62.5 (SE 3.2) Low load: 0.0 (SE 3.2) CT: -18.5 (SE 3.1) High versus CT: *p* = 0.02 Others *p* = N.S. Overall *p* = 0.042		None	Lower limb volume was significantly reduced after high-load AECT compared to CT. Change in symptoms score were similar across the three interventions	Volume: low risk
										Pain/heaviness: high risk
**Sierakowski, Piller** **2014** **Australia**	A = 9 D = 0	**Volume** + compression Mean change: 136 (SE 50) ml Difference between sessions: 28 (SE 60) ml, *p* = 0.62			No compression Mean change: 174 (SE 57) ml			None	SC may provide a socially acceptable and effective means of lymphedema control during exercise for early-stage lower limb LE as measured by bioimpedance, but not confirmed by perometry	Volume: some concerns
**Wittenkamp et. al** **2024** **Denmark** ^1^	A = 19 D = 2	**Pain** + compression Mean change -0.1 [95% CI -0.4; 0.3] No compression Mean change -0.4 [95% CI -0.8; -0.1] Difference between sessions: 0.4 [95% CI -0.1; 0.8] *p* = 0.09		**Heaviness** + compression Mean change -0.2 [95% CI -0.8; 0.5] No compression Mean change -0.2 [95% CI -0.9; 0.6] Difference between sessions: 0.0 [95% CI -0.8; 0.8] *p* = 1.00			**Volume** + compression Mean change 65.3 ml [95% CI -24.6; 155.22] No compression Mean change 83.9 ml [95% CI -13.4; 181.3] Difference between sessions: 18.7 ml [95% CI -150.2; 112.8] *p* = 0.8	None	HIIT on a stationary bike was acceptable for patients with LLL and seemed safe regardless of the use of compressions garments	Pain/heaviness: low risk
										Volume: low risk

Values are mean (SD), unless otherwise indicated.

* Data reported as median (minimum-maximum), but calculated to mean (SD); 6MWT: 6 Minute Walk Test; A: Analyzed; AECT: Active Exercise with Compression Therapy; ALT: Aqua Lymphatic Therapy; C: Control Group; CDT: Complete Decongestive Therapy; CG: Control Group; CT: Compression-only Therapy; D: dropout; IG: Intervention Group; HOOS: Hip Osteoarthritis Outcome Score; HR-QOL: Health-Related Quality of Life; IG: Intervention Group; ISL: International Society of Lymphology; LE: Lymphedema; N.S.: Not Significant; SC: Sporting Compression; SD: Standard Deviation.

1 Manuscript in review: Wittenkamp MC, Juhl, C.B., Zerahn, B., Vinther, A. Exercise and cancer-related lymphedema in the lower limbs - a randomized cross-over trial on High Intensity Interval Training (HIIT) with and without compression garments

**Table 3:** Summary of findings.

Exercise for patients with lower limb lymphedema				
**Population**: Patients with lower limb lymphedema **Settings**: Hospital/rehabilitation center/home-based **Intervention**: Exercise (single component or multi-component intervention (e.g. compression therapy, manual lymphatic drainage, skin care) **Comparison**: No exercise/another type of exercise/standard care/patient education*				
Outcome	Participants	Risk of bias	Certainty of evidence (GRADE)	Importance of outcome

Quality of Life	166 (7 trials)	Serious risk of bias	Very low (+000)	Important
Self-reported symptoms (pain or heaviness)	106 (4 trials)	Serious risk of bias	Very low (+000)	Important
Physical function	141 (5 trials)	Serious risk of bias	Very low (+000)	Important
Limb volume	238 (11 trials)	Serious risk of bias	Very low (+000)	Not important

Effect sizes were not calculated due to large heterogeneity among trials with different study design.				
*RCT trials only. Study design varied with 3 RCT, 5 single group studies, and 4 crossover trials.				

RCT: Randomized controlled trial.

**Table 4:** Ongoing trials identified at Clinicaltrials.gov.

Trial Identifier Design	Investigator Country	Title	Diagnosis	Sample size *n*	Age sex	Intervention Type Duration	Primary Outcome	Secondary Outcome	Study Status
NCT05483569 RCT	Acar Turkey	Diaphragmatic Exercises and Fascial Release Techniques on the Treatment of Lower Extremity Lymphedema	LLL Gynecological cancer	30	25–65 women	2 groups 1) CDT + placebo fascial release techniques 2) CDT + multidimensional diaphragmatic breathing exercises and fascial release techniques 3 weeks	Limb volume Functional status Sleep quality	Passive tone Stiffness Creep Relaxation	Unknown status
NCT06327412 RCT	Çalış. Turkey	The Effects of Aerobic Exercise in Patients with Primary Lower Extremity Lymphedema	Primary LLL, Stage 2–3	35	18–65	2 groups 1) Aerobic exercise on the treadmill, group-based 2) Walking between 12 and 13 RPE, homebased 4 weeks	30s-CST	6MWT Limb Volume BIS LEFS LLIC HADS Pain, NRS	Not yet recruiting
NCT06545383 RCT	Çiftçi Turkey	The Effect of Dynamic Neuromuscular Stabilization Exercises in Patients with Lower Extremity Lymphedema	Primary or Secondary LLL	42	18+ years	2 groups 1) Dynamic Neuromuscular Stabilization Exercises 2) Home-based breathing exercises + joint movement exercise + walking 6 weeks	Static balance on one leg dynamic balance	Joint Position Sense 10MWT Functional status Fear of falling Limb Volume	Not yet recruiting
NCT05003973 RCT	Hsu Taiwan	The Effectiveness of Anti-resistance Exercise on Lower Limb Lymphoedema Among Gynecological Cancers	LLL, Gynecological cancer	100	20–75 women	2 groups 1) Home-based, anti-resistance exercise 2) Usual care, leaflet with tips for care for LLL 12 weeks	Limb circumference BIS Muscle strength Fatigue HR-QOL		Unknown status
NCT06200948 RCT	Kara Turkey	Effects of Aerobic Cycling Training in Patients with Gynecologic Cancer-related Lower Extremity Lymphedema	LLL, Gynecological cancer	63	18–65 women	2 groups 1) CDT + bike ergometer exercise 2) CDT 3 weeks	HR-QOL Limb volume Functional status		Completed
NCT05609526 RCT	Mazı Turkey	Inspiratory and Calf Muscles Training in Patients with Leg Lymphedema	LLL	45	18–75 years	4 groups 1) CB 2) Inspiratory muscle training +CB 3) Calf muscle exercise +CB 4) All interventions combined 4 weeks	HR-QOL Limb volume	6MWT Leg fullness NRS TDC	Unknown status

6MWT: 6 Minute Walk Test; 10MWT: 10 Meters Walking Test; 30s-CST: 30 seconds Chair Stand Test; BIS: BioImpedance Spectroscopy; CB: Compression bandaging; CDT: Complete Decongestive Therapy; HADS: Hospital Depression and Anxiety Scale; HR-QOL: Health-Related Quality of Life; LEFS: Lower Extremity Functional Scale; LLL: Lower Limb Lymphedema; LLIC: Lymphedema Life Impact Scale; NRS: Numerical Rating Scale; RPE: The Borg Rating of Perceived Exertion; TDC: Tissue Dielectric Constant; RCT: Randomized controlled trial.

### Exercise interventions

Interventions differed across studies: water-based exercise [18, 20, 23], aerobic exercise using indoor stationary bike or outdoor biking [25, 26, 28]^1^ or walking on a treadmill [27], resistance exercise [24], or a mix of aerobic and strength exercises as an add-on to CDT [19, 21, 22]. Control groups (CGs) in the RCTs received CDT [19] or Self-MLD combined with land-based exercises [18] or no exercise intervention [28], respectively. Apart from two trials [22, 28], exercise interventions were supervised [18, 20, 21, 23, 25–27] ^1^ or partly supervised [19, 24] across RCTs and single group studies.

Exercise was performed at moderate intensity in three trials [19, 23, 28] and with progressive intensity in three trials [24, 25, 27]. Duration of the exercise interventions in the RCTs and single group studies varied between 3 and 6 weeks [18, 19, 21–23], 8 and 10 weeks [20, 28], and 5 months [24] with an exercise frequency of 3–6 times per week [19, 21, 22, 28], twice a week [18, 23, 24], and once a week [20] (Table 1).

In the crossover trials, exercises were combined with the application of compression bandaging (CB) [25, 26] and custom-made compression garments^1^ or sports compression tights [27] compared to an equal session without compression. Exercise was performed on an indoor stationary bike or outdoor bike [25, 26]^1^ or by walking on a treadmill with an increase of speed or incline every 3 min until 85% of maximal heart rate was reached following a submaximal exercise test protocol [27].

### Outcomes

#### Health-related quality of life

HR-QOL was assessed in three RCTs and three single group studies with five different outcome measures: European Organization for Research and Treatment of Cancer Quality of Life Questionnaire C30 (EORTC-QLQ-C30) [19, 22], Short Form-36 (SF-36) [18], Lymphedema Quality of Life Inventory (LyQLI) [28], Lymphedema Quality of Life Tool (LyMQOL) [23], and the Freiburg Quality of Life Assessment for lymphatic disorders, short version (FLQA-lk) [21] (Table 1).

Overall HR-QOL did not improve statistically significant in the RCTs [18, 19, 28], but Do et al. reported significant larger improvements in the dimensions: physical function and fatigue [19] and likewise in several subscales of the SF-36 by Ergin et al. [18] (Table 2). Among single group studies, Zeng et al. also reported statistically significant improvements across subscales [22]. Statistically significant improvement in overall HR-QOL was reported by Angst et al. and Dionne et al. [21, 23], but not the remaining trials [20, 24].

#### Physical function

**Figure 2:**
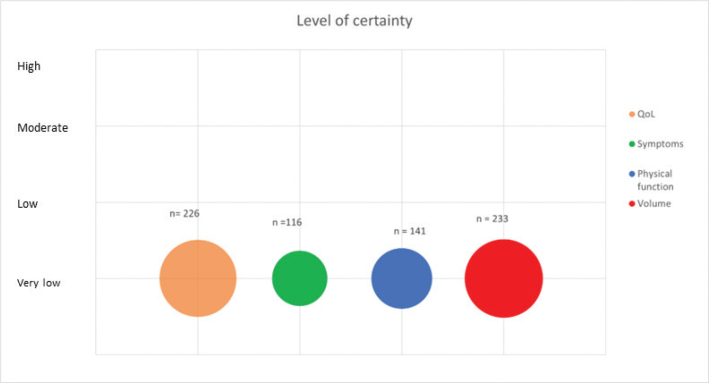
Level of certainty across outcomes. Bubble plot of level of certainty across outcomes. Level of certainty is depicted at the Y-axis. The size of each bubble reflects the number of participants measured for each outcome across the included trials.

Physical function was assessed in three RCTs and two single group studies with different outcome measures: 6 minute Walk-Test (6MWT) [18, 23, 24], submaximal VO_2_ max test [28], 30 seconds Chair Stand test (30s-CST) [19], or 50-feet walk time (50ft WT)(24) (Table 2 and Figure 2). Do et al. reported a statistically significant improvement in 30s-CST by 2.4 repetitions (*p* = 0.001) in the IG [19]. Neither Ergin et al. nor Jönsson et al. found any difference between groups in 6MWT [18] or VO_2_ max [28], respectively. Statistically significant improvements in 6MWT were seen in the single group study by Dionne et al., 61.6 m, *p* = 0.04 [23], and Katz et al., 29 m, *p* ≤ 0.01 and by 10.5 m (*p* ≤ 0.01) in 50ft WT [24]).

#### Symptoms

Self-reported pain and heaviness were assessed in one single group study and in three cross-over trials [22, 25, 26]^1^ (Table 2). Zeng et al. reported significant improvements in both outcomes measured by the 20-item Gynecologic Tumor Lymphedema Questionnaire [22]. In the crossover trial by Abe et al., a statistically significant larger mean reduction in pain and heaviness was seen in supine exercise compared to seated exercise: pain, 71.0% versus 27.9% (*p* = 0.02); heaviness, 62.3% versus 27.5% (*p* = 0.03) [25]. Additionally, high- or low-load exercise with CB versus resting with CB showed a larger reduction in self-reported pain (VAS 32.8/24.1 vs^.^ 12.0) and heaviness (VAS 47.2/43.4 vs. 33.5), respectively, but change scores were not statistically significant [26]. Likewise, change scores in pain and heaviness were not statistically significant in the trial by Wittenkamp et al. (*p* = 0.09 and *p* = 1.0)^1^.

#### Lower limb volume

Lower limb volumes were reported in all trials except for the single group study by Angst et al. [21] (Table 1). In the RCTs, no significant difference in limb volume between groups was found [18, 19, 28] (Table 2). Among single group studies, Dionne et al. reported a statistically significant decrease in lower limb volume (844.4 ml, *p* = 0.03) [23], and Zeng et al. found a summed decrease of both limbs of 53.3 cm (*p* = 0.00) [22]. The other single group studies did not find a statistically significant decrease in limb volume post-exercise [20, 24]. In the cross-over trials, lower limb volume decreased with high-load exercise and CB compared to CB only (62.5 vs. 18.5, *p* = 0.02) [26] and with exercise in supine position compared to seated position (71% vs. 45.4%, *p* = 0.01) [25]. The remaining cross-over trials did not achieve significant decrease between exercise with and without sports compression tights [27] or compression garments^1^.

### Adverse events

Eight trials assessed adverse events [18, 21, 24–28]^1^. Three adverse events were reported in two trials: one participant experienced an increase in lower limb volume >5% and was referred to CDT [28], and in the trial with resistance exercise, two out of 10 participants developed cellulitis [24] (Table 2).

### Risk of bias

In RCTs, blinding of participants was not performed, and blinding of assessor was only reported in Ergin et al. and Jönsson et al. [18, 28]. The selection of participants was not clearly described in the RCTs and single group studies, resulting in a high risk of selection bias (Table 2). In the single group studies, adjustments for important factors, for example adherence to intervention, use of CDT, and change in physical activity levels during the intervention period, were not assessed or discussed, which increased the risk of bias in the results presented. Overall risk of bias on the selected outcomes was rated as moderate or high in most RCTs and all single group studies and one of the cross-over trials [18–24, 27]. The crossover trials with bike ergometer were rated as low risk of bias due to good methodology and precise reporting [25, 26]^1^ (Table 2).

### Certainty of evidence (GRADE)

The included trials were characterized by high risk of bias and large heterogeneity across trials with participants with cancer-related LE and primary LE, different exercise modalities, exercise frequencies, and outcome measures. There is a low number of participants overall, and publication bias cannot be ruled out. Across all outcomes, certainty of evidence was considered very low (Table 3 and Figure 2).

## Discussion

This systematic review aimed to synthesize the immediate and long-term effect of exercise for patients with LLL. It is the first review including patients across primary or secondary LLL, whereas previous reviews have focused on patients with LLL after gynecological cancer [9] or patients with BCRL [7, 8, 29, 30]. Our review cannot present a meta-analysis on the effect of exercise for patients with LLL due to a small number of exercise studies with large heterogeneity including different outcome measures, poor methodological quality, and low certainty of evidence. However, irrespective of severity, exercise seemed to have small but positive effects on HR-QOL, physical function, self-reported pain and heaviness, and lower limb volume for patients with LLL. The number of adverse events in the trials in this review were limited with three cases among a total 300 participants across studies. However, it should be noted that four trials did not report on adverse events, illustrating that uncertainty about adverse events in exercise for patients with LLL still remains.

The results of this review are identical to the results of the review of exercise for patients with LLL after gynaecological cancer by Hsu et al. on the outcomes of self-reported symptoms and limb volume, although the studies included in each review are different due to different eligibility criteria [9]. Both reviews have summarized data on the exercise modalities: water-based exercise, aerobic exercise, and one study with weightlifting. In water-based exercise, the hydrostatic pressure may provide an identical effect to the lymphatic system as compression garments, but no significant effect in limb volume was seen in the trials, which may be due to only low to moderate intensity exercise.

15-Min-high-load aerobic exercise or exercise in supine position on a bike combined with the newly applied compression bandages showed significant reductions in limb volume in the trials by Fukushima et al. and Abe et al., respectively [25, 26], showing a potential for immediate edema reduction and alleviation of self-reported symptoms. However, the application of bandages before exercise and biking in supine position may not be applicable at home for patients with LLL. Aerobic exercise with compression garments or sports compression tights at moderate to high intensity was assessed in the trials by Jønsson et al. [28]), Wittenkamp et al.^1^, and Sierakowski [27], but compression did not achieve significant immediate or long-term reductions in limb volume, which may be explained by insufficient pressure from compression garments and tights.

Exercise guidelines from ACSM and World Health Organization (WHO) recommend that adults engage in 150–300 min of moderate-intensity physical activity combined with two weekly sessions of muscle strengthening activities, and the recommendations also apply to patients with LE [5, 31]. Previously, progressive resistance exercise has been proved safe for patients at risk of or diagnosed with BCRL [5, 7]. It is noteworthy that only one pilot study with resistance exercise [24] was identified for the present review, and that none of the ongoing studies (Table 4) investigates the effect of resistance exercise illustrating the lack of knowledge about the safety and benefits for this exercise modality for patients with LLL in particular. Across the longitudinal trials, notable improvements in HR-QOL were observed in three out of seven studies [18, 22, 23] and in physical function in three out of five studies [19, 23, 24]. Although the sample sizes were small, these findings remain important for patients with LLL, as LE can contribute to physical impairments, emotional distress, and reduced HR-QOL.

In conclusion, exercise has several health benefits, but most importantly, physical activity and movement can increase the flow in the lymphatic system and support the lymph transport [4]. Therefore, exercise should a part of successful self-management for patients with LLL. Additionally, physical activity may be helpful for weight management or even weight loss as obesity itself can impair the lymphatic transport leading to poorer prognosis for obese patients with LE with increased risk of infections and hospitalizations [32, 33]. However, patients with LLL may experience fear of movement [34, 35], which must be taken into consideration in self-management programs with exercise included for patients with LLL.

Our review has been performed according to recommendations from the Cochrane Handbook of systematic reviews, registered at PROSPERO, and is reported according to The Preferred Reporting Items for Systematic Reviews and Meta-Analyses guidelines [36], which strengthens the work, but the review has obvious limitations due to the lack of high-quality RCTs, no meta-analysis conducted, and the inclusion of single group studies and crossover trials in the review. Including trials with multicomponent interventions, for example exercise combined with CDT or Intermittent Pneumatic Compression (IPC) [19, 21, 22] in the review, makes it difficult to establish the exact effect of exercise in the trials, which is also an obvious limitation in the review.

Hsu et al. did perform a meta-analysis on the randomized cross-over trials by Abe et al. [25] and Fukushima et al. [26]. Despite the inclusion of three RCTs in this review, meta-analysis was not considered appropriate due to the risk of an unreliable result and limited statistical power in the analysis.

## Implications for clinical practice and future research

Exercise should be encouraged as a part of management of LLL despite no existing evidence on modality, intensity, and delivery specifically for patients with LLL. For safety, exercise may be delivered as supervised, slow progressive exercise at first with both aerobic and resistance modalities with the monitoring of self-reported symptoms and LE assessment, for example limb volume.

Obviously, there is a need of high-quality RCTs to establish the impact of exercise for patients with LLL across different exercise modalities, exercise intensity, and delivery modes. We suggest that single modality trials with either aerobic and resistance exercise interventions should be performed, and that exercise interventions should meet exercise recommendations from the ACSM and the WHO [5, 37]. Additionally, exercise interventions should be applicable to a home setting for successful implementation, for example walking or biking interventions or resistance exercise with simple equipment or easy accessible in the immediate surroundings. Future studies should obviously be performed with randomization and blinding of assessors to reduce the risk of bias. In 2023, a Core Outcome Measure (COM) for the assessment of BCRL was developed [38]. A similar COM for the assessment of LLL could potentially strengthen the design of exercise trials for LLL and thereby improve the possibility of synthesizing evidence on exercise for patients with LLL. Until a COM for LLL is developed, several outcome measures in the COM for BCRL may also be relevant to patients with LLL, for example HR-QOL, self-reported symptoms, and limb volume. Adverse events such as skin irritation, cellulitis, wounds, and muscle injuries should also be assessed due to the limited research in exercise and adverse events in LLL.

## Conclusion

Due to the large variation in the exercise interventions and small sample size with overall poor research methodology, it was not possible to make firm conclusions regarding which exercise modalities and exercise intensities will benefit patients with LLL the most. This conclusion is consistent with a recent review on exercise for patients with LLL after gynecological cancer [9] and a review from 2022 on exercise for patients with cancer-related LE, which mainly included trials on patients with upper limb LE, which likewise failed to deliver conclusions on exercise for patients with LLL [10]. However, this review could help in the design of exercise trials for patients with LLL.

## Supplementary Material



## Footnotes

1 Manuscript in review: Wittenkamp MC, Juhl, C.B., Zerahn, B., Vinther, A. Exercise and cancer-related lymphedema in the lower limbs - a randomized cross-over trial on High Intensity Interval Training (HIIT) with and without compression garments

## Registration

This systematic review was registered at PROSPERO CRD42022340176.

## References

[1] Rockson SG, Keeley V, Kilbreath S, Szuba A, Towers A. Cancer-associated secondary lymphoedema. Nat Rev Dis Primers. 2019;5(1):22. 10.1038/s41572-019-0072-5 30923312

[2] Best Practice for the Management of Lymphoedema. International consensus. London: MEP Ltd; 2006.

[3] Document C. The diagnosis and treatment of peripheral Lymphedema: 2020 consensus document of the international society of Lymphology. Lymphology. 2020;53:3–19. 10.2458/lymph.4649 32521126

[4] Mortimer PS, Pearson M, Gawrysiak P, Riches K, Keeley V, Tew KF, et al. LymphActiv: a digital physical activity behavior intervention for the treatment of lymphedema and lipedema. Lymphatic Res Biol. 2024;22(2):112–9. 10.1089/lrb.2023.0033 38394133

[5] Campbell KL, Winters-Stone KM, Wiskemann J, May AM, Schwartz AL, Courneya KS, et al. Exercise guidelines for cancer survivors: consensus statement from international multidisciplinary roundtable. Med Sci Sports Exerc. 2019;51(11):2375–2390. 10.1249/MSS.0000000000002116 31626055 PMC8576825

[6] Meiklejohn J, Heesch K, Janda M, Hayes S. Physical activity in the lives of those living with lymphoedema following cancer treatment. Lymphology. 2012;44(Suppl):131–7.

[7] Hayes SC, Singh B, Reul-Hirche H, Bloomquist K, Johansson K, Jönsson C, et al. The effect of exercise for the prevention and treatment of cancer-related lymphedema: a systematic review with meta-analysis. Med Sci Sports Exerc. 2022;54(8):1389–99. 10.1249/MSS.0000000000002918 35320145

[8] Singh B, Disipio T, Peake J, Hayes SC. Systematic review and meta-analysis of the effects of exercise for those with cancer-related lymphedema. Arch Phys Med Rehabil. 2016;97(2):302–15.e13. 10.1016/j.apmr.2015.09.012 26440777

[9] Hsu Y-Y, Nguyen TTB, Chou Y-J, Ho C-L. Effects of exercise on lower limb lymphedema in gynecologic cancer: a systematic review and meta-analysis. Eur J Oncol Nurs. 2024;70:102550. 10.1016/j.ejon.2024.102550 38554614

[10] DiSipio T, Rye S, Newman B, Hayes S. Incidence of unilateral arm lymphoedema after breast cancer: a systematic review and meta-analysis. Lancet Oncol. 2013;14(6):500–15. 10.1016/S1470-2045(13)70076-7 23540561

[11] Cormier JN, Askew RL, Mungovan KS, Xing Y, Ross MI, Armer JM. Lymphedema beyond breast cancer: a systematic review and meta-analysis of cancer-related secondary lymphedema. Cancer. 2010;116(22):5138–49. 10.1002/cncr.25458 20665892

[12] Higgins JPT, Thomas J, Chandler J, Cumpston M, Li T, Page MJ, Welch VA (editors). Cochrane Handbook for Systematic Reviews of Interventions version 6.5 (updated August 2024). Cochrane, 2024. Available from www.training.cochrane.org/handbook.

[13] Meiklejohn JA, Heesch KC, Janda M, Hayes SC. How people construct their experience of living with secondary lymphoedema in the context of their everyday lives in Australia. Support Care Cancer. 2013;21(2):459–66. 10.1007/s00520-012-1534-4 23010957

[14] Sterne JAC, Savović J, Page MJ, Elbers RG, Blencowe NS, Boutron I, et al. RoB 2: a revised tool for assessing risk of bias in randomised trials. BMJ. 2019;366:l4898. 10.1136/bmj.l4898 31462531

[15] Revised Cochrane risk-of-bias tool for randomized crossover trials 2021. [updated 2021 March 18]. [cited date 2024 July 20] Available from: https://www.riskofbias.info/welcome/rob-2-0-tool/rob-2-for-crossover-trials

[16] Sterne JA, Hernán MA, Reeves BC, Savović J, Berkman ND, Viswanathan M, et al. ROBINS-I: a tool for assessing risk of bias in non-randomised studies of interventions. BMJ. 2016;355:i4919. 10.1136/bmj.i4919 27733354 PMC5062054

[17] Guyatt GH, Oxman AD, Vist GE, Kunz R, Falck-Ytter Y, Alonso-Coello P, et al. GRADE: an emerging consensus on rating quality of evidence and strength of recommendations. BMJ. 2008;336(7650):924–6. 10.1136/bmj.39489.470347.AD 18436948 PMC2335261

[18] Ergin G, Karadibak D, Sener HO, Gurpinar B. Effects of aqua-lymphatic therapy on lower extremity lymphedema: a randomized controlled study. Lymphatic Res Biol. 2017;15(3):284–91. 10.1089/lrb.2017.0017 28880750

[19] Do JH, Choi KH, Ahn JS, Jeon JY. Effects of a complex rehabilitation program on edema status, physical function, and quality of life in lower-limb lymphedema after gynecological cancer surgery. Gynecol Oncol. 2017;147(2):450–5. 10.1016/j.ygyno.2017.09.003 28941657

[20] Lindquist H, Enblom A, Dunberger G, Nyberg T, Bergmark K. Water exercise compared to land exercise or standard care in female cancer survivors with secondary lymphedema. Lymphology. 2015;48(2):64–79. 26714371

[21] Angst F, Benz T, Lehmann S, Sándor PS, Wagner S. Effects of inpatient rehabilitation in leg lymphedema: a naturalistic prospective cohort study with intra-individual control of effects. Arch Phys Med Rehabil. 2023;104(12):2035–42. 10.1016/j.apmr.2023.06.001 37329968

[22] Zeng Y, Liu G, Peng Z, Hu J, Zhang A. Application of complete decongestive therapy in patients with secondary bilateral lower limb lymphedema after comprehensive treatment of gynecological malignant tumor. Lymphat Res Biol. 2024;22(1):60–5. 10.1089/lrb.2023.0029 37787968

[23] Dionne A, Goulet S, Leone M, Comtois AS. Aquatic exercise training outcomes on functional capacity, quality of life, and lower limb lymphedema: pilot study. J Altern Complement Med. 2018;24(9–10):1007–9. 10.1089/acm.2018.0041 30247973

[24] Katz E, Dugan NL, Cohn JC, Chu C, Smith RG, Schmitz KH. Weight lifting in patients with lower-extremity lymphedema secondary to cancer: a pilot and feasibility study. Arch Phys Med Rehabil. 2010;91(7):1070–6. 10.1016/j.apmr.2010.03.021 20599045 PMC2897812

[25] Abe K, Tsuji T, Oka A, Shoji J, Kamisako M, Hohri H, et al. Postural differences in the immediate effects of active exercise with compression therapy on lower limb lymphedema. Support Care Cancer. 2021;29(11):6535–43. 10.1007/s00520-020-05976-y 33928435 PMC8464559

[26] Fukushima T, Tsuji T, Sano Y, Miyata C, Kamisako M, Hohri H, et al. Immediate effects of active exercise with compression therapy on lower-limb lymphedema. Support Care Cancer. 2017;25(8):2603–10. 10.1007/s00520-017-3671-2 28386788 PMC5486768

[27] Sierakowski K, Piller N. Pilot study of the impact of sporting compression garments on composition and volume of normal and lymphedema legs. Lymphology. 2014;47(4):187–95. 25915979

[28] Jönsson CJ, Bjurberg K, Brogårdh, MC. Lower limb lymphedema: evaluation of measurements and exercise. Faculty of Medicine: Lund University, Lund; 2023.

[29] Paramanandam VS, Roberts D. Weight training is not harmful for women with breast cancer-related lymphoedema: a systematic review. J Physiother. 2014;60(3):136–43. 10.1016/j.jphys.2014.07.001 25086730

[30] Yeung W, Semciw AI. Aquatic therapy for people with lymphedema: a systematic review and meta-analysis. Lymphat Res Biol. 2018;16(1):9–19. 10.1089/lrb.2016.0056 28346851

[31] WHO guidelines on physical activity and sedentary behaviour. Geneva: World Health Organization; 2020. Licence: CC BY-NC-SA 3.0 IGO. 10.1136/bjsports-2020-102955 PMC7719906 33239350

[32] Burian EA, Rungby J, Karlsmark T, Nørregaard S, Cestari M, Franks PJ, et al. The impact of obesity on chronic oedema/lymphoedema of the leg – an international multicenter cross-sectional study (LIMPRINT). Int J Obes (Lond). 2024;48(9):1238–47. 10.1038/s41366-024-01544-0 38961152 PMC11347371

[33] Greene AK, Zurakowski D, Goss JA. Body mass index and lymphedema morbidity: comparison of obese versus normal-weight patients. Plast Reconstr Surg. 2020;146(2):402–7. 10.1097/PRS.0000000000007021 32740596

[34] Sahbaz Pirincci C, Cihan E, Borman P, Dalyan M. Does Fear of movement affect fatigue and quality of life in lower extremity lymphedema? Lymphat Res Biol. 2023;21(3):270–4. 10.1089/lrb.2022.0050 36580543

[35] Pirincci CS, Cihan E, Ünüvar BS, Gerçek H, Aytar A, Borman P. Investigation of physical activity, fear of falling, and functionality in individuals with lower extremity lymphedema. Support Care Cancer. 2023;31(6):360. 10.1007/s00520-023-07825-0 37247048

[36] Page MJ, McKenzie JE, Bossuyt PM, Boutron I, Hoffmann TC, Mulrow CD, et al. The PRISMA 2020 statement: an updated guideline for reporting systematic reviews. BMJ. 2021;372:n71. 10.1136/bmj.n71 33782057 PMC8005924

[37] Bull FC, Al-Ansari SS, Biddle S, Borodulin K, Buman MP, Cardon G, et al. World Health Organization 2020 guidelines on physical activity and sedentary behaviour. Br J Sports Med. 2020;54(24):1451–62. 10.1136/bjsports-2020-102955 33239350 PMC7719906

[38] Doubblestein D, Koehler L, Anderson E, Scheiman N, Stewart P, Schaverien M, et al. Development of a core set of outcome measures to be applied toward breast cancer-related lymphedema core outcome domains. Breast Cancer Res Treat. 2024;205(3):439–49. 10.1007/s10549-024-07298-7 38517603 PMC11101581

