# Supplementary material for: The effect of exercise in patients with lower limb lymphedema: a systematic review
Source: Acta Oncol. 2025 Mar 31;64:42560. doi: 10.2340/1651-226X.2025.42560 (PMC11977414; doi:10.2340/1651-226X.2025.42560)
Supplement: Supplementary file 1 [file AO-64-42560-s1.pdf]

## Appendix 1 Search from MEDLINE

(((Lymphedema[mh] OR Lymphoedema [tiab] OR Lymphedema[tiab]) AND (leg[TIAB] OR leg [MeSH] OR Lower limb[TIAB]) OR "Lower limb lymph *" [tiab] OR "Leg lymph*" [tiab])) AND (((((((((((((((((((((((((((((((((exercise[MeSH Terms]) OR (exercise*[Title/Abstract])) OR (exercise therapy[MeSH Terms])) OR ("exercise therap*"[Title/Abstract])) OR ("physical activit*"[Title/Abstract])) OR (modalities, physical therapy[MeSH Terms])) OR (exercise movement techniques[MeSH Terms])) OR ("exercise movement technique"[Title/Abstract])) OR (sports[MeSH Terms])) OR (sport*[Title/Abstract])) OR ("Physical Fitness"[Title/Abstract])) OR ("Cardiovascular exercis*"[Title/Abstract])) OR ("Cardiovascular train*"[Title/Abstract])) OR ("aerobic exercise*"[Title/Abstract])) OR ("endurance training"[Title/Abstract])) OR ("muscle train*"[Title/Abstract])) OR ("muscle strength*"[Title/Abstract])) OR ("strength train*"[Title/Abstract])) OR ("resistance train*"[Title/Abstract])) OR ("weight lift*"[Title/Abstract])) OR (runnin*[Title/Abstract])) OR (Joggin*[Title/Abstract])) OR ("step activit*"[Title/Abstract])) OR (Walking[Title/Abstract])) OR ("water exercis*"[Title/Abstract])) OR ("water based exercis*"[Title/Abstract])) OR ("aquatic exercis*"[Title/Abstract])) OR ("high intensity interval train*"[Title/Abstract])) OR ("interval train*"[Title/Abstract])) OR (yoga[Title/Abstract])) OR (pilates[Title/Abstract])) OR ("tai chi"[Title/Abstract])) OR (Dance*[Title/Abstract]))

## Appendix 2 Search Strategy Embase

1. lymphedema/
2. lymphoedema.mp. [mp=title, abstract, heading word, drug trade name, original title, device manufacturer, drug manufacturer, device trade name, keyword heading word, floating subheading word, candidate term word]
3. lymphedema.mp. [mp=title, abstract, heading word, drug trade name, original title, device manufacturer, drug manufacturer, device trade name, keyword heading word, floating subheading word, candidate term word]
4. leg.mp. [mp=title, abstract, heading word, drug trade name, original title, device manufacturer, drug manufacturer, device trade name, keyword heading word, floating subheading word, candidate term word]
5. lower limb.mp. [mp=title, abstract, heading word, drug trade name, original title, device manufacturer, drug manufacturer, device trade name, keyword heading word, floating subheading word, candidate term word]
6. lower limb lymph*.mp. [mp=title, abstract, heading word, drug trade name, original title, device manufacturer, drug manufacturer, device trade name, keyword heading word, floating subheading word, candidate term word]
7. leg lymph*.mp. [mp=title, abstract, heading word, drug trade name, original title, device manufacturer, drug manufacturer, device trade name, keyword heading word, floating subheading word, candidate term word]
8. 1 or 2 or 3
9. 4 or 5 or 6 or 7
10. 8 and 9
11. exercise/
12. exercise*.mp.
13. exercise therap*.mp.
14. kinesiotherapy/
15. physical activity/
16. physical activity.mp.
17. exercise movement techniq*.mp.
18. sport/
19. sport*.mp.
20. fitness/
21. physical fitness.mp.
22. aerobic exercise/

23. cardiovascular exercis*.mp.
24. cardiovascular train*.mp.
25. endurance training/
26. endurance train*.mp.
27. muscle training/
28. muscle train*.mp.
29. muscle strength/
30. muscle strength*.mp.
31. strenght train*.mp.
32. resistance training/
33. resistance train*.mp.
34. weight lifting/
35. weight lift*.mp.
36. running/
37. runnin*.mp.
38. jogging/
39. jogging.mp.
40. step activit*.mp.
41. walking/
42. walking.mp.
43. water exercis*.mp.
44. water based exercis*.mp.
45. aquatic exercise/
46. aquatic exercis*.mp.
47. high intensity interval training/
48. high intensity interval train*.mp.
49. interval train*.mp.

50. yoga/
51. yoga.mp.
52. pilates/
53. pilates.mp.
54. Tai Chi/
55. tai chi.mp.
56. dancing/
57. dance.mp.
58. 11 or 12 or 13 or 14 or 15 or 16 or 17 or 18 or 19 or 20 or 21 or 22 or 23 or 24 or 25 or 26 or 27 or 28 or 29 or 30 or 31 or 32 or 33 or 34 or 35 or 36 or 37 or 38 or 39 or 40 or 41 or 42 or 43 or 44 or 45 or 46 or 47 or 48 or 49 or 50 or 51 or 52 or 53 or 54 or 55 or 56 or 57
59. 10 and 58

| # | Query | Limiters/Expanders | Last Run Via | Results |
|---|---|---|---|---|
| S79 | S16 AND S78 | Expanders - Apply equivalent subjects<br>Search modes - Proximity | Interface - EBSCOhost Research Databases<br>Search Screen - Advanced Search<br>Database - CINAHL | Display |
| S78 | (S17 OR S18 OR S19 OR S20 OR S21 OR S22 OR S23 OR S24 OR S25 OR S26 OR S27 OR S28 OR S29 OR S30 OR S31 OR S32 OR S33 OR S34 OR S35 OR S36 OR S37 OR S38 OR S39 OR S40 OR S41 OR S42 OR S43 OR S44 OR S45 OR S46 OR S47 OR S48 OR S49 OR S50 OR S51 OR S52 OR S53 OR S54 OR S55 OR S56 OR S57 OR S58 OR S59 OR S60 OR S61 OR S62 OR S63 OR S64 OR S65 OR S66 OR S67 OR S68 OR S69 OR S70 OR S71 OR S72 OR S73 OR S74 OR S75 OR S76 OR S77) | Expanders - Apply equivalent subjects<br>Search modes - Proximity | Interface - EBSCOhost Research Databases<br>Search Screen - Advanced Search<br>Database - CINAHL | Display |
| S77 | AB dance* | Expanders - Apply equivalent subjects<br>Search modes - Proximity | Interface - EBSCOhost Research Databases<br>Search Screen - Advanced Search<br>Database - CINAHL | Display |
| S76 | TI dance* | Expanders - Apply equivalent subjects<br>Search modes - Proximity | Interface - EBSCOhost Research Databases<br>Search Screen - Advanced Search<br>Database - CINAHL | Display |
| S75 | AB "tai chi" | Expanders - Apply equivalent subjects<br>Search modes - Proximity | Interface - EBSCOhost Research Databases<br>Search Screen - Advanced Search<br>Database - CINAHL | Display |
| S74 | TI "tai chi" | Expanders - Apply equivalent subjects<br>Search modes - Proximity | Interface - EBSCOhost Research Databases<br>Search Screen - Advanced Search<br>Database - CINAHL | Display |
| S73 | AB pilates | Expanders - Apply equivalent subjects<br>Search modes - Proximity | Interface - EBSCOhost Research Databases<br>Search Screen - Advanced Search<br>Database - CINAHL | Display |
| S72 | TI pilates | Expanders - Apply equivalent subjects<br>Search modes - Proximity | Interface - EBSCOhost Research Databases<br>Search Screen - Advanced Search<br>Database - CINAHL | Display |
| S71 | AB yoga | Expanders - Apply equivalent subjects<br>Search modes - Proximity | Interface - EBSCOhost Research Databases<br>Search Screen - Advanced Search<br>Database - CINAHL | Display |

| | | | | |
|---|---|---|---|---|
| S70 | TI yoga | Expanders - Apply equivalent subjects<br>Search modes - Proximity | Interface - EBSCOhost Research Databases<br>Search Screen - Advanced Search<br>Database - CINAHL | Display |
| S69 | AB "interval train*" | Expanders - Apply equivalent subjects<br>Search modes - Proximity | Interface - EBSCOhost Research Databases<br>Search Screen - Advanced Search<br>Database - CINAHL | Display |
| S68 | TI "interval train*" | Expanders - Apply equivalent subjects<br>Search modes - Proximity | Interface - EBSCOhost Research Databases<br>Search Screen - Advanced Search<br>Database - CINAHL | Display |
| S67 | AB "high intensity interval train*" | Expanders - Apply equivalent subjects<br>Search modes - Proximity | Interface - EBSCOhost Research Databases<br>Search Screen - Advanced Search<br>Database - CINAHL | Display |
| S66 | TI "high intensity interval train*" | Expanders - Apply equivalent subjects<br>Search modes - Proximity | Interface - EBSCOhost Research Databases<br>Search Screen - Advanced Search<br>Database - CINAHL | Display |
| S65 | AB "aquatic exercis*" | Expanders - Apply equivalent subjects<br>Search modes - Proximity | Interface - EBSCOhost Research Databases<br>Search Screen - Advanced Search<br>Database - CINAHL | Display |
| S64 | TI "aquatic exercis*" | Expanders - Apply equivalent subjects<br>Search modes - Proximity | Interface - EBSCOhost Research Databases<br>Search Screen - Advanced Search<br>Database - CINAHL | Display |
| S63 | AB "water based exercis*" | Expanders - Apply equivalent subjects<br>Search modes - Proximity | Interface - EBSCOhost Research Databases<br>Search Screen - Advanced Search<br>Database - CINAHL | Display |
| S62 | TI "water based exercis*" | Expanders - Apply equivalent subjects<br>Search modes - Proximity | Interface - EBSCOhost Research Databases<br>Search Screen - Advanced Search<br>Database - CINAHL | Display |
| S61 | AB "water exercis*" | Expanders - Apply equivalent subjects<br>Search modes - Proximity | Interface - EBSCOhost Research Databases<br>Search Screen - Advanced Search<br>Database - CINAHL | Display |
| S60 | TI "water exercis*" | Expanders - Apply equivalent subjects<br>Search modes - Proximity | Interface - EBSCOhost Research Databases<br>Search Screen - Advanced Search<br>Database - CINAHL | Display |
| S59 | AB walkin* | Expanders - Apply equivalent subjects<br>Search modes - Proximity | Interface - EBSCOhost Research Databases<br>Search Screen - Advanced Search<br>Database - CINAHL | Display |
| S58 | TI walkin* | Expanders - Apply equivalent subjects<br>Search modes - Proximity | Interface - EBSCOhost Research Databases<br>Search Screen - Advanced Search<br>Database - CINAHL | Display |
| S57 | AB "step activit*" | Expanders - Apply equivalent subjects<br>Search modes - Proximity | Interface - EBSCOhost Research Databases<br>Search Screen - Advanced Search<br>Database - CINAHL | Display |

| | | | | |
|---|---|---|---|---|
| S56 | TI "step activit*" | Expanders - Apply equivalent subjects<br>Search modes - Proximity | Interface - EBSCOhost Research Databases<br>Search Screen - Advanced Search<br>Database - CINAHL | Display |
| S55 | AB joggin* | Expanders - Apply equivalent subjects<br>Search modes - Proximity | Interface - EBSCOhost Research Databases<br>Search Screen - Advanced Search<br>Database - CINAHL | Display |
| S54 | TI joggin* | Expanders - Apply equivalent subjects<br>Search modes - Proximity | Interface - EBSCOhost Research Databases<br>Search Screen - Advanced Search<br>Database - CINAHL | Display |
| S53 | AB runnin* | Expanders - Apply equivalent subjects<br>Search modes - Proximity | Interface - EBSCOhost Research Databases<br>Search Screen - Advanced Search<br>Database - CINAHL | Display |
| S52 | TI runnin* | Expanders - Apply equivalent subjects<br>Search modes - Proximity | Interface - EBSCOhost Research Databases<br>Search Screen - Advanced Search<br>Database - CINAHL | Display |
| S51 | AB "weight lift*" | Expanders - Apply equivalent subjects<br>Search modes - Proximity | Interface - EBSCOhost Research Databases<br>Search Screen - Advanced Search<br>Database - CINAHL | Display |
| S50 | TI "weight lift*" | Expanders - Apply equivalent subjects<br>Search modes - Proximity | Interface - EBSCOhost Research Databases<br>Search Screen - Advanced Search<br>Database - CINAHL | Display |
| S49 | AB "resistance train*" | Expanders - Apply equivalent subjects<br>Search modes - Proximity | Interface - EBSCOhost Research Databases<br>Search Screen - Advanced Search<br>Database - CINAHL | Display |
| S48 | TI "resistance train*" | Expanders - Apply equivalent subjects<br>Search modes - Proximity | Interface - EBSCOhost Research Databases<br>Search Screen - Advanced Search<br>Database - CINAHL | Display |
| S47 | AB "strength train*" | Expanders - Apply equivalent subjects<br>Search modes - Proximity | Interface - EBSCOhost Research Databases<br>Search Screen - Advanced Search<br>Database - CINAHL | Display |
| S46 | TI "strength train*" | Expanders - Apply equivalent subjects<br>Search modes - Proximity | Interface - EBSCOhost Research Databases<br>Search Screen - Advanced Search<br>Database - CINAHL | Display |
| S45 | AB "muscle strength*" | Expanders - Apply equivalent subjects<br>Search modes - Proximity | Interface - EBSCOhost Research Databases<br>Search Screen - Advanced Search<br>Database - CINAHL | Display |
| S44 | TI "muscle strength*" | Expanders - Apply equivalent subjects<br>Search modes - Proximity | Interface - EBSCOhost Research Databases<br>Search Screen - Advanced Search<br>Database - CINAHL | Display |
| S43 | AB "muscle train*" | Expanders - Apply equivalent subjects<br>Search modes - Proximity | Interface - EBSCOhost Research Databases<br>Search Screen - Advanced Search<br>Database - CINAHL | Display |

| | | | | |
|---|---|---|---|---|
| S42 | TI "muscle train*" | Expanders - Apply equivalent subjects<br>Search modes - Proximity | Interface - EBSCOhost Research Databases<br>Search Screen - Advanced Search<br>Database - CINAHL | Display |
| S41 | AB "endurance train*" | Expanders - Apply equivalent subjects<br>Search modes - Proximity | Interface - EBSCOhost Research Databases<br>Search Screen - Advanced Search<br>Database - CINAHL | Display |
| S40 | TI "endurance train*" | Expanders - Apply equivalent subjects<br>Search modes - Proximity | Interface - EBSCOhost Research Databases<br>Search Screen - Advanced Search<br>Database - CINAHL | Display |
| S39 | AB "aerobic exercis*" | Expanders - Apply equivalent subjects<br>Search modes - Proximity | Interface - EBSCOhost Research Databases<br>Search Screen - Advanced Search<br>Database - CINAHL | Display |
| S38 | TI "aerobic exercis*" | Expanders - Apply equivalent subjects<br>Search modes - Proximity | Interface - EBSCOhost Research Databases<br>Search Screen - Advanced Search<br>Database - CINAHL | Display |
| S37 | AB "cardiovascular train*" | Expanders - Apply equivalent subjects<br>Search modes - Proximity | Interface - EBSCOhost Research Databases<br>Search Screen - Advanced Search<br>Database - CINAHL | Display |
| S36 | TI "cardiovascular train*" | Expanders - Apply equivalent subjects<br>Search modes - Proximity | Interface - EBSCOhost Research Databases<br>Search Screen - Advanced Search<br>Database - CINAHL | Display |
| S35 | AB "cardiovascular exercis*" | Expanders - Apply equivalent subjects<br>Search modes - Proximity | Interface - EBSCOhost Research Databases<br>Search Screen - Advanced Search<br>Database - CINAHL | Display |
| S34 | TI "cardiovascular exercis*" | Expanders - Apply equivalent subjects<br>Search modes - Proximity | Interface - EBSCOhost Research Databases<br>Search Screen - Advanced Search<br>Database - CINAHL | Display |
| S33 | AB "physical fitness" | Expanders - Apply equivalent subjects<br>Search modes - Proximity | Interface - EBSCOhost Research Databases<br>Search Screen - Advanced Search<br>Database - CINAHL | Display |
| S32 | TI "physical fitness" | Expanders - Apply equivalent subjects<br>Search modes - Proximity | Interface - EBSCOhost Research Databases<br>Search Screen - Advanced Search<br>Database - CINAHL | Display |
| S31 | AB sport* | Expanders - Apply equivalent subjects<br>Search modes - Proximity | Interface - EBSCOhost Research Databases<br>Search Screen - Advanced Search<br>Database - CINAHL | Display |
| S30 | TI sport* | Expanders - Apply equivalent subjects<br>Search modes - Proximity | Interface - EBSCOhost Research Databases<br>Search Screen - Advanced Search<br>Database - CINAHL | Display |
| S29 | MH sports | Expanders - Apply equivalent subjects<br>Search modes - Proximity | Interface - EBSCOhost Research Databases<br>Search Screen - Advanced Search<br>Database - CINAHL | Display |

| | | | | |
|---|---|---|---|---|
| S28 | AB "exercise movement techniques" | Expanders - Apply equivalent subjects<br>Search modes - Proximity | Interface - EBSCOhost Research Databases<br>Search Screen - Advanced Search<br>Database - CINAHL | Display |
| S27 | TI "exercise movement techniques" | Expanders - Apply equivalent subjects<br>Search modes - Proximity | Interface - EBSCOhost Research Databases<br>Search Screen - Advanced Search<br>Database - CINAHL | Display |
| S26 | MH exercise movement techniques" | Expanders - Apply equivalent subjects<br>Search modes - Proximity | Interface - EBSCOhost Research Databases<br>Search Screen - Advanced Search<br>Database - CINAHL | Display |
| S25 | MH "physical activity modalities" | Expanders - Apply equivalent subjects<br>Search modes - Proximity | Interface - EBSCOhost Research Databases<br>Search Screen - Advanced Search<br>Database - CINAHL | Display |
| S24 | AB "physical activit*" | Expanders - Apply equivalent subjects<br>Search modes - Proximity | Interface - EBSCOhost Research Databases<br>Search Screen - Advanced Search<br>Database - CINAHL | Display |
| S23 | TI "physical activit*" | Expanders - Apply equivalent subjects<br>Search modes - Proximity | Interface - EBSCOhost Research Databases<br>Search Screen - Advanced Search<br>Database - CINAHL | Display |
| S22 | MH "physical activity" | Expanders - Apply equivalent subjects<br>Search modes - Proximity | Interface - EBSCOhost Research Databases<br>Search Screen - Advanced Search<br>Database - CINAHL | Display |
| S21 | AB "exercise therap*" | Expanders - Apply equivalent subjects<br>Search modes - Proximity | Interface - EBSCOhost Research Databases<br>Search Screen - Advanced Search<br>Database - CINAHL | Display |
| S20 | TI "exercise therap*" | Expanders - Apply equivalent subjects<br>Search modes - Proximity | Interface - EBSCOhost Research Databases<br>Search Screen - Advanced Search<br>Database - CINAHL | Display |
| S19 | AB exercise | Expanders - Apply equivalent subjects<br>Search modes - Proximity | Interface - EBSCOhost Research Databases<br>Search Screen - Advanced Search<br>Database - CINAHL | Display |
| S18 | TI exercise | Expanders - Apply equivalent subjects<br>Search modes - Proximity | Interface - EBSCOhost Research Databases<br>Search Screen - Advanced Search<br>Database - CINAHL | Display |
| S17 | MH exercise | Expanders - Apply equivalent subjects<br>Search modes - Proximity | Interface - EBSCOhost Research Databases<br>Search Screen - Advanced Search<br>Database - CINAHL | Display |
| S16 | S6 AND S15 | Expanders - Apply equivalent subjects<br>Search modes - Proximity | Interface - EBSCOhost Research Databases<br>Search Screen - Advanced Search<br>Database - CINAHL | Display |
| S15 | S7 OR S8 OR S9 OR S10 OR S11 OR S12 OR S13 OR S14 | Expanders - Apply equivalent subjects<br>Search modes - Proximity | Interface - EBSCOhost Research Databases<br>Search Screen - Advanced Search<br>Database - CINAHL | Display |

| | | | | |
|---|---|---|---|---|
| S14 | AB "leg lymph*" | Expanders - Apply equivalent subjects<br>Search modes - Proximity | Interface - EBSCOhost Research Databases<br>Search Screen - Advanced Search<br>Database - CINAHL | Display |
| S13 | TI "leg lymph*" | Expanders - Apply equivalent subjects<br>Search modes - Proximity | Interface - EBSCOhost Research Databases<br>Search Screen - Advanced Search<br>Database - CINAHL | Display |
| S12 | AB "lower limb lymph*" | Expanders - Apply equivalent subjects<br>Search modes - Proximity | Interface - EBSCOhost Research Databases<br>Search Screen - Advanced Search<br>Database - CINAHL | Display |
| S11 | TI "lower limb lymph*" | Expanders - Apply equivalent subjects<br>Search modes - Proximity | Interface - EBSCOhost Research Databases<br>Search Screen - Advanced Search<br>Database - CINAHL | Display |
| S10 | AB "lower limb" | Expanders - Apply equivalent subjects<br>Search modes - Proximity | Interface - EBSCOhost Research Databases<br>Search Screen - Advanced Search<br>Database - CINAHL | Display |
| S9 | TI "lower limb" | Expanders - Apply equivalent subjects<br>Search modes - Proximity | Interface - EBSCOhost Research Databases<br>Search Screen - Advanced Search<br>Database - CINAHL | Display |
| S8 | AB leg | Expanders - Apply equivalent subjects<br>Search modes - Proximity | Interface - EBSCOhost Research Databases<br>Search Screen - Advanced Search<br>Database - CINAHL | Display |
| S7 | TI leg | Expanders - Apply equivalent subjects<br>Search modes - Proximity | Interface - EBSCOhost Research Databases<br>Search Screen - Advanced Search<br>Database - CINAHL | Display |
| S6 | S1 OR S2 OR S3 OR S4 OR S5 | Expanders - Apply equivalent subjects<br>Search modes - Proximity | Interface - EBSCOhost Research Databases<br>Search Screen - Advanced Search<br>Database - CINAHL | Display |
| S5 | AB lymphoedema | Expanders - Apply equivalent subjects<br>Search modes - Proximity | Interface - EBSCOhost Research Databases<br>Search Screen - Advanced Search<br>Database - CINAHL | Display |
| S4 | TI lymphoedema | Expanders - Apply equivalent subjects<br>Search modes - Proximity | Interface - EBSCOhost Research Databases<br>Search Screen - Advanced Search<br>Database - CINAHL | Display |
| S3 | AB lymphedema | Expanders - Apply equivalent subjects<br>Search modes - Proximity | Interface - EBSCOhost Research Databases<br>Search Screen - Advanced Search<br>Database - CINAHL | Display |
| S2 | TI lymphedema | Expanders - Apply equivalent subjects<br>Search modes - Proximity | Interface - EBSCOhost Research Databases<br>Search Screen - Advanced Search<br>Database - CINAHL | Display |
| S1 | MH lymphedema | Expanders - Apply equivalent subjects<br>Search modes - Proximity | Interface - EBSCOhost Research Databases<br>Search Screen - Advanced Search<br>Database - CINAHL | Display |

# PRISMA 2020 Main Checklist

| | | | |
|---|---|---|---|
| **TITLE** | | | |
| Title | 1 | Identify the report as a systematic review. | Page 1 |
| **ABSTRACT** | | | |
| Abstract | 2 | See the PRISMA 2020 for Abstracts checklist | |
| **INTRODUCTION** | | | |
| Rationale | 3 | Describe the rationale for the review in the context of existing knowledge. | Page 3, section 2 |
| Objectives | 4 | Provide an explicit statement of the objective(s) or question(s) the review addresses. | Page 3, section 2 |
| **METHODS** | | | |
| Eligibility criteria | 5 | Specify the inclusion and exclusion criteria for the review and how studies were grouped for the syntheses. | page 4, section 2 |
| Information sources | 6 | Specify all databases, registers, websites, organisations, reference lists and other sources searched or consulted to identify studies. Specify the date when each source was last searched or consulted. | Page 3, section 4 |
| Search strategy | 7 | Present the full search strategies for all databases, registers and websites, including any filters and limits used. | Appendix 1-4 |
| Selection process | 8 | Specify the methods used to decide whether a study met the inclusion criteria of the review, including how many reviewers screened each record and each report retrieved, whether they worked independently, and if applicable, details of automation tools used in the process. | page 4, section 4 |

*(continued)*

| | | | |
|---|---|---|---|
| Data collection process | 9 | Specify the methods used to collect data from reports, including how many reviewers collected data from each report, whether they worked independently, any processes for obtaining or confirming data from study investigators, and if applicable, details of automation tools used in the process. | Page 5, section 1 |
| Data items | 10a | List and define all outcomes for which data were sought. Specify whether all results that were compatible with each outcome domain in each study were sought (e.g. for all measures, time points, analyses), and if not, the methods used to decide which results to collect. | page 4, section 2 |
| | 10b | List and define all other variables for which data were sought (e.g. participant and intervention characteristics, funding sources). Describe any assumptions made about any missing or unclear information. | not applicable |
| Study risk of bias assessment | 11 | Specify the methods used to assess risk of bias in the included studies, including details of the tool(s) used, how many reviewers assessed each study and whether they worked independently, and if applicable, details of automation tools used in the process. | page 5, section 2 |
| Effect measures | 12 | Specify for each outcome the effect measure(s) (e.g. risk ratio, mean difference) used in the synthesis or presentation of results. | Not applicable |
| Synthesis methods | 13a | Describe the processes used to decide which studies were eligible for each synthesis (e.g. tabulating the study intervention characteristics and comparing against the planned groups for each synthesis (item 5)). | not applicable |

*(continued)*

| | | | |
|---|---|---|---|
| | 13b | Describe any methods required to prepare the data for presentation or synthesis, such as handling of missing summary statistics, or data conversions. | Not applicable |
| | 13c | Describe any methods used to tabulate or visually display results of individual studies and syntheses. | not applicable |
| | 13d | Describe any methods used to synthesize results and provide a rationale for the choice(s). If meta-analysis was performed, describe the model(s), method(s) to identify the presence and extent of statistical heterogeneity, and software package(s) used. | Page 5, section 3 |
| | 13e | Describe any methods used to explore possible causes of heterogeneity among study results (e.g. subgroup analysis, meta-regression). | Not applicable |
| | 13f | Describe any sensitivity analyses conducted to assess robustness of the synthesized results. | Not applicable |
| Reporting bias assessment | 14 | Describe any methods used to assess risk of bias due to missing results in a synthesis (arising from reporting biases). | not applicable |
| Certainty assessment | 15 | Describe any methods used to assess certainty (or confidence) in the body of evidence for an outcome. | page 5, section 3 |
| **RESULTS** | | | |
| Study selection | 16a | Describe the results of the search and selection process, from the number of records identified in the search to the number of studies included in the review, ideally using a flow diagram. | page 5, section 4 and figure 1 |
| | 16b | Cite studies that might appear to meet the inclusion criteria, but which were excluded, and explain why they were excluded. | Not applicable |
| Study characteristics | 17 | Cite each included study and present its characteristics. | page 5, section 4 and table 1 |

*(continued)*

| | | | |
|---|---|---|---|
| Risk of bias in studies | 18 | Present assessments of risk of bias for each included study. | page8, section 2, Appendix 6-8 and table 2 |
| Results of individual studies | 19 | For all outcomes, present, for each study: (a) summary statistics for each group (where appropriate) and (b) an effect estimate and its precision (e.g. confidence/credible interval), ideally using structured tables or plots. | table 2 and figure 2 |
| Results of syntheses | 20a | For each synthesis, briefly summarise the characteristics and risk of bias among contributing studies. | not applicable |
| | 20b | Present results of all statistical syntheses conducted. If meta-analysis was done, present for each the summary estimate and its precision (e.g. confidence/credible interval) and measures of statistical heterogeneity. If comparing groups, describe the direction of the effect. | Not applicable |
| | 20c | Present results of all investigations of possible causes of heterogeneity among study results. | Not applicable |
| | 20d | Present results of all sensitivity analyses conducted to assess the robustness of the synthesized results. | Not applicable |
| Reporting biases | 21 | Present assessments of risk of bias due to missing results (arising from reporting biases) for each synthesis assessed. | Not applicable |
| Certainty of evidence | 22 | Present assessments of certainty (or confidence) in the body of evidence for each outcome assessed. | table 3 and figure 3 |
| **DISCUSSION** | | | |
| Discussion | 23a | Provide a general interpretation of the results in the context of other evidence. | page 9 |
| | 23b | Discuss any limitations of the evidence included in the review. | page 11 |
| | 23c | Discuss any limitations of the review processes used. | page 10, section 3 |

*(continued)*

| | | | |
|---|---|---|---|
| | 23d | Discuss implications of the results for practice, policy, and future research. | page 10, section 4 |
| **OTHER INFORMATION** | | | |
| Registration and protocol | 24a | Provide registration information for the review, including register name and registration number, or state that the review was not registered. | page 14 |
| | 24b | Indicate where the review protocol can be accessed, or state that a protocol was not prepared. | page 14 |
| | 24c | Describe and explain any amendments to information provided at registration or in the protocol. | Not applicable |
| Support | 25 | Describe sources of financial or non-financial support for the review, and the role of the funders or sponsors in the review. | page 14 |
| Competing interests | 26 | Declare any competing interests of review authors. | page 14 |
| Availability of data, code and other materials | 27 | Report which of the following are publicly available and where they can be found: template data collection forms; data extracted from included studies; data used for all analyses; analytic code; any other materials used in the review. | Not applicable |

## PRIMSA Abstract Checklist

| | | | |
|---|---|---|---|
| **TITLE** | | | |
| Title | 1 | Identify the report as a systematic review. | Yes |
| **BACKGROUND** | | | |
| Objectives | 2 | Provide an explicit statement of the main objective(s) or question(s) the review addresses. | Yes |
| **METHODS** | | | |
| Eligibility criteria | 3 | Specify the inclusion and exclusion criteria for the review. | Yes |
| Information sources | 4 | Specify the information sources (e.g. databases, registers) used to identify studies and the date when each was last searched. | Yes |
| Risk of bias | 5 | Specify the methods used to assess risk of bias in the included studies. | Yes |
| Synthesis of results | 6 | Specify the methods used to present and synthesize results. | Yes |
| **RESULTS** | | | |
| Included studies | 7 | Give the total number of included studies and participants and summarise relevant characteristics of studies. | Yes |
| Synthesis of results | 8 | Present results for main outcomes, preferably indicating the number of included studies and participants for each. If meta-analysis was done, report the summary estimate and confidence/credible interval. If comparing groups, indicate the direction of the effect (i.e. which group is favoured). | Yes |
| **DISCUSSION** | | | |
| Limitations of evidence | 9 | Provide a brief summary of the limitations of the evidence included in the review (e.g. study risk of bias, inconsistency and imprecision). | Yes |
| Interpretation | 10 | Provide a general interpretation of the results and important implications. | Yes |
| **OTHER** | | | |
| Funding | 11 | Specify the primary source of funding for the review. | No |
| Registration | 12 | Provide the register name and registration number. | No |

*From:* Page MJ, McKenzie JE, Bossuyt PM, Boutron I, Hoffmann TC, Mulrow CD, et al. The PRISMA 2020 statement: an updated guideline for reporting systematic reviews. MetaArXiv. 2020, September 14. DOI: 10.31222/osf.io/v7gm2. For more information, visit: www.prisma-statement.org

# Appendix 6 Risk of Bias rating of Randomized controlled trials

| Intention-to-treat | Unique ID | Study ID | Experimental | Comparator | Outcome | Weight | D1 | D2 | D3 | D4 | D5 | Overall |
|---|---|---|---|---|---|---|---|---|---|---|---|---|
| | DO_HR QOL | Do_HRQOL | CDT + exercise | CDT only | HRQOL | 1 | ! | ! | ! | ! | ! | ! |
| | DO_volume | Do_volume | CDT + exercise | CDT only | volume | 1 | ! | ! | ! | ! | - | - |
| | DO_pain | Do_pain | CDT + exercise | CDT only | Physical function | 1 | ! | + | ! | ! | - | - |
| | Ergin_HRQOL | Ergin_HRQOL | Aquatic exercise + selfc | Selfdrainage | HRQOL | 1 | + | - | ! | ! | ! | - |
| | Ergin_volume | Ergin_volume | Aquatic exercise + selfc | Selfdrainage | volume | 1 | + | - | ! | + | - | - |
| | Ergin_6MWT | Ergin_6MWT | Aquatic exercise | Self-drainage | 6MWT | 1 | + | - | ! | + | ! | - |
| | Jønsson | Jønsson HR-QOL | Biking | Usual care | HR-QOL | 1 | ! | - | - | ! | + | - |
| | Jønsson- volu | Jønsson volume | Biking | Usual care | Volume | 1 | ! | + | - | + | + | - |
| | Jønsson. Phys | Jønsson Physical f | Biking | Usual care | Jønsson Physical func | 1 | ! | + | - | + | + | - |

+ Low risk

! Some concerns

- High risk

D1 Randomisation process

D2 Deviations from the intended interventions

D3 Missing outcome data

D4 Measurement of the outcome

D5 Selection of the reported result

## Appendix 7 Risk of Bias Non-Randomized Controlled Trials

| Study | Intervention | Outcome | D1 | D2 | D3 | D4 | D5 | D6 | D7 | Overall |
|---|---|---|---|---|---|---|---|---|---|---|
| **Angst** | Aqua Exercise + CDT | HR-QOL | ! | + | ! | + | - | ! | + | - |
| **Dionne** | Aqua Exercise | HR-QOL | - | - | ! | ! | + | ! | ! | - |
| | | Physical function | - | - | ! | ! | + | ! | ! | - |
| | | Volume | - | - | ! | ! | + | + | ! | - |
| **Katz** | Resistance Exercise | HR-QOL | - | + | ! | ! | + | - | ! | - |
| | | Physical function | - | + | ! | ! | + | - | ! | - |
| | | Volume | - | + | ! | ! | + | - | ! | - |
| **Lindquist** | Aqua Exercise | Volume | ! | ! | + | + | ! | ! | ! | - |
| **Zeng** | CDT + IPC | HR-QOL | - | - | ! | + | ! | ! | ! | - |
| | | Symptoms | - | - | ! | + | ! | ! | ! | - |
| | | Volume | - | - | ! | + | - | ! | ! | - |

+ low risk of rias

! moderate risk of bias

\- high risk of bias

D1 Confounding

D2 Selection of participants into the study

D3 Classification of interventions

D4 Deviations from intended interventions

D5 Missing data

D6 Measurement of outcomes

D7 Selection of reported results

6M WT: 6 minute Walk Test, CDT: Complete Decongestive Therapy, HR-HRL: Health-related Quality of Life, IPC: Intermittent Pneumatic Compression. VAS: Visual Analog Scale,

# Appendix 8 Risk of Bias rating in Cross-over trials

| Unique ID | Study ID | Experimental | Comparator | Outcome | Weight | D1 | DS | D2 | D3 | D4 | D5 | Overall |
|---|---|---|---|---|---|---|---|---|---|---|---|---|
| Abe_volume | Abe_volume | Seated/supine exercise | Elevation/no exercise | volume | 1 | + | + | + | + | + | + | + |
| Abe_selfreport | Abe_selfreport | Seated/supine exercise | Elevation/no exercise | selfreported pain and tension | 1 | + | + | + | + | + | + | + |
| fukushima_volume | Fukushima_volume | High/low load exercise | No exercise | volume | 1 | + | + | + | + | + | + | + |
| fukushima_selfreport | Fukushima_selfreport | High/low load exercise | No exercise | selfreported pain and tension | 1 | + | + | + | + | + | + | + |
| sierakowski_volume | sierakowski_volume | compression thights | no compression | volume | 1 | - | - | ! | + | + | ! | - |
| wittenkamp_pain | wittenkamp_pain | Compression | no compression | Selfreported pain | 1 | + | + | + | + | + | + | + |
| wittenkamp_volume | wittenkamp_volume | compression | no compression | Volume | 1 | + | + | + | + | + | + | + |

+ Low risk

! Some concerns

- High risk

D1 Randomisation process

DS Bias arising from period and carryover effects

D2 Deviations from the intended interventions

D3 Missing outcome data

D4 Measurement of the outcome

D5 Selection of the reported result
